# Influence of Post-Weld Heat Treatment on Mechanical Properties and Microstructure of Plasma Arc-Welded 316 Stainless Steel

**DOI:** 10.3390/ma17153768

**Published:** 2024-07-31

**Authors:** Adirek Baisukhan, Nirut Naksuk, Pinmanee Insua, Wasawat Nakkiew, Nuttachat Wisittipanit

**Affiliations:** 1 Department of Industrial Engineering, Faculty of Engineering, Chiang Mai University, Chiang Mai 50200, Thailand; adirek.b@cmu.ac.th (A.B.); pmn.insua@gmail.com (P.I.); wasawat@eng.cmu.ac.th (W.N.); 2 National Metal and Materials Technology Center (MTEC), National Science and Technology Development Agency, Pathum Thani 12120, Thailand; 3 Department of Materials Engineering, School of Science, Mae Fah Luang University, Chiang Rai 57100, Thailand; nuttachat.wis@mfu.ac.th

**Keywords:** plasma arc welding, post-weld heat treatment, 316 stainless steel, industry innovation and infrastructure

## Abstract

This study investigates the effects of post-weld heat treatment (PWHT) on the microstructures and mechanical properties of plasma arc-welded 316 stainless steel. The experimental parameters included the solid solution temperatures of 650 °C and 1050 °C, solid solution durations of 1 h and 4 h, and quenching media of water and air. The mechanical properties were evaluated using Vickers hardness testing, tensile testing, scanning electron microscopy (SEM), and optical microscopy (OM). The highest ultimate tensile strength (UTS) of 693.93 MPa and Vickers hardness of 196.4 in the welded zone were achieved by heat-treating at 650 °C for one hour, quenching in water, and aging at 500 °C for 24 h. Heat-treating at 650 °C for one hour, followed by quenching in water and aging at 500 °C for 24 h results in larger dendritic δ grains and contains more σ phase compared to the other conditions, resulting in increased strength and hardness. Additionally, it shows wider and shallower dimple structures, which account for its reduced impact toughness.

## 1. Introduction

Stainless steel continues to be the most sought-after material [1] in numerous manufacturing industries, primarily due to its combination of strength and corrosion resistance, which provides a significant advantage over inexpensive and readily available carbon steel. Furthermore, its affordability and availability position it as the preferred choice over other corrosion-resistant alloys [2]. Among the various grades of stainless steel, austenitic stainless steel represents the largest family of alloys and exhibits extensive industrial applications. This is attributed to its superior service performance and low carbon content, facilitating easier fabrication processes, particularly welding [3]. Within the austenitic stainless steels, grade 316 is recognized as the standard molybdenum-bearing grade, second in importance only to grade 304. The presence of molybdenum endows grade 316 with enhanced overall corrosion resistance compared to grade 304, especially in chloride environments where pitting and crevice corrosion are prevalent. Additionally, grade 316 possesses excellent forming and welding properties.

Plasma arc welding (PAW) is an advanced arc welding technique wherein metals are joined by heating them with a constricted arc formed between a tungsten electrode and the metals being welded [4,5]. The generation of a high-velocity plasma in PAW is facilitated by utilizing a non-consumable tungsten electrode and an inert gas, which serves to shield the molten weld pool. This process bears a close resemblance to gas tungsten arc welding (GTAW), also known as tungsten inert gas (TIG) welding, as both employ an arc as the heat source for fusing joints, with the option of adding filler metal when necessary [6,7]. One of the key advantages of PAW over GTAW is its ability to produce higher quality welds while being more cost-effective compared to other high-energy welding methods such as laser beam or electron beam welding. This makes PAW potentially the most efficient method for a wide range of applications [8]. Unlike TIG welding, PAW minimizes heat loss due to the smaller surface area of the arc exposed to air, thereby enhancing its efficiency [9]. PAW is characterized by superior weld penetration, reduced angular distortion and residual stress, and the prevention of grain overgrowth, all of which contribute to its enhanced efficiency compared to other conventional arc welding methods [10]. However, the heat input from the PAW process can lead to certain degradation issues in the microstructural and mechanical properties of the welded material. These issues include microstructural inhomogeneity, residual stress concentration, increased brittleness, and reduced toughness [11]. Extensive research has been conducted on PAW to elucidate its effects from various perspectives. The primary focus of these studies has been on optimizing PAW process parameters to enhance weld efficiency and quality for different materials. Research topics have included weld bead geometry [12,13], welding speed [14], arc pressure [15,16], weld penetration [13,14,17], hardness, and ultimate tensile strength [18,19]. Among the materials studied, aluminum alloys [20,21] and stainless steel [8,13,22,23,24,25,26,27] are particularly notable. Additionally, various techniques have been employed during or after the PAW process to further improve weld efficiency in terms of mechanical properties. Post-weld heat treatment (PWHT) is frequently employed to mitigate such deterioration [28]. Appropriate PWHT can enhance mechanical characteristics by homogenizing the microstructure of the welded material and preventing the formation of unfavorable phases. However, few studies have investigated the effects of PWHT on plasma arc-welded workpieces. Karaoğlu and Kaçar [29] explored the impact of heat treatment on the properties of TRIP800 steel fabricated by PAW. Furthermore, a study by Kuril et al. [30] indicated that most tensile residual stresses are alleviated by the PWHT process. Nevertheless, 316 stainless steel has not been extensively studied for weldment quality improvement through PWHT, from both properties and structural perspectives.

PAW is widely employed in space industries [31] and has been identified as a more capable welding method for critical components in the aircraft and automotive industries [10]. The development in the post-process stage is crucial for enhancing mechanical properties. This study aims to contribute to the existing knowledge on improving material properties, particularly for stainless steel components. Therefore, this investigation seeks to present the effects of PWHT on the microstructural and mechanical qualities of the welded sections of PAW’s 316 stainless steel, serving as a preliminary study for future research in the PAW field. The novelty of this study lies in the application of PWHT to 316 stainless steel welded using PAW. The objectives of this study are as follows:

- To identify the optimal process parameters for the post-weld heat treatment of 316 stainless steel welded using PAW.
- To enhance the mechanical properties of 316 stainless steel welded using PAW through the application of post-weld heat treatment.

## 2. Materials and Methods

### 2.1. Workpiece Preparation

**Figure 1:**
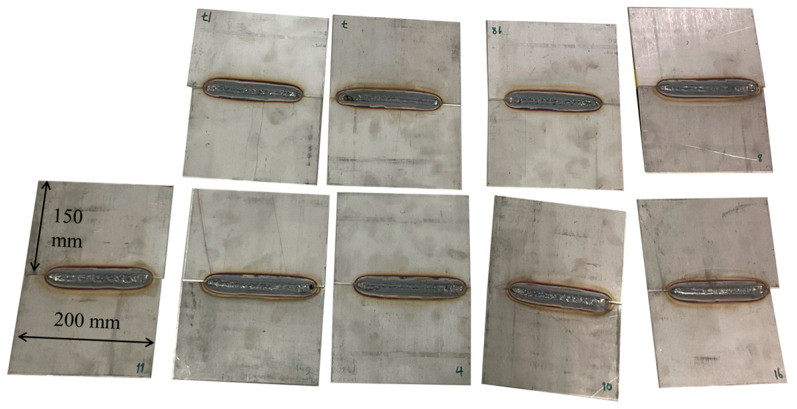
Samples of 316 Stainless steel plates welded by plasma arc welding.

316 austenitic stainless-steel plates, with dimensions of 200 mm in length, 150 mm in width, and 3 mm in thickness, were used in this study. The chemical composition measured using the energy-dispersive X-ray fluorescence (EDXRF) method is shown in Table 1. Nine coupled plates were welded using the plasma arc welding process under the same conditions, as shown in Figure 1, for further processing.

### 2.2. Post-Weld Heat Treatment (PWHT)

Multiple conditions utilized in the PWHT process are presented in Table 2. The condition values obtained from a literature review were selected to design a full factorial experiment with three factors and two levels, as illustrated in Table 3. The PWHT process began with heating a sample at the solid solution treatment temperatures of 650 °C and 1050 °C for the experimental times of 1 h and 4 h. Subsequently, the sample was quenched in different media (water and air). Finally, the sample was aged at a temperature of 500 °C for 24 h [32,33]. The different steps in PWHT process applied in this study as shown in Table 4.

### 2.3. Tensile Test

**Figure 2:**
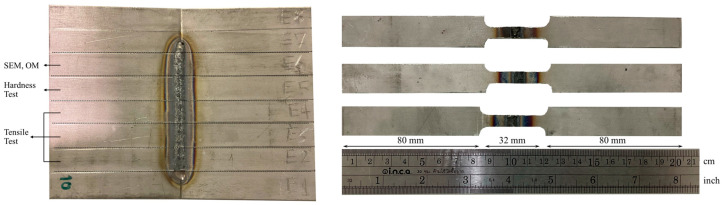
An example of a sample cut into the shape of a dog bone.

**Figure 3:**
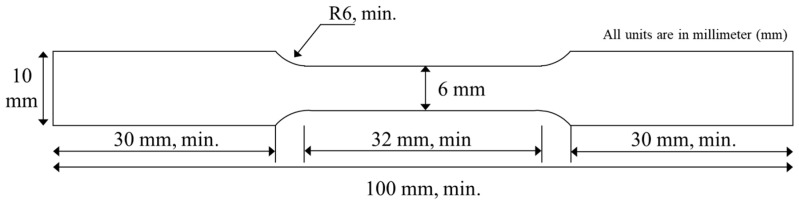
A dog bone size of ASTM E8 specification.

After undergoing the PWHT process, all the samples were prepared for mechanical properties testing. For the tensile test, the samples were cut into the standard dog bone shape in accordance with ASTM E8 specifications, as shown in Figure 2 and Figure 3. Three specimens were tested for each condition. The tensile test was conducted using a universal tensile machine (MTS, Eden Prairie, MN, USA) with a strain rate of 10^−1^/s.

### 2.4. Vickers Hardness Test

**Figure 4:**
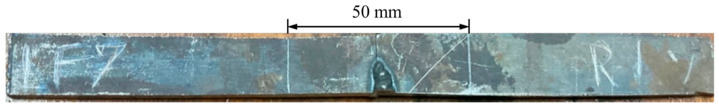
Area of interest for Vickers hardness evaluation.

**Figure 5:**
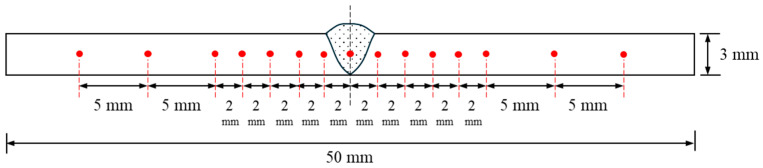
Specific points on the specimen for Vickers hardness evaluation.

The Vickers hardness test measures a material’s property by calculating the hardness number from the load over the surface area of the indentation. When performing a hardness test, an initial force and the duration of the force are required. Firstly, the samples were sectioned into 50 mm lengths, as shown in Figure 4. The cut sections were then molded using a mixture of epoxy resin and hardening agent in a 2:1 ratio. Subsequently, sandpapers with grit sizes of 90, 120, 600, 800, and 1200 were used to smooth the cross-sectional surfaces. Final polishing was performed using a diamond paste. Evaluation points were marked on the polished samples, as illustrated in Figure 5, to prepare for testing. The Vickers hardness test was conducted with a load of 2.94 N and a dwell time of 15 s. The hardness values were measured ten times per sample, and the average value was calculated by excluding the maximum and minimum values.

### 2.5. Microstructure Evaluation

**Figure 6:**
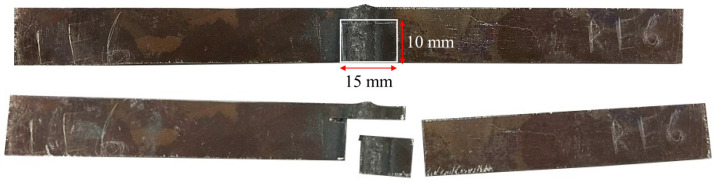
A cutting part for microstructure evaluation.

The samples were cut to a size of 15 mm in length and 10 mm in width, positioning the welded zone on the left side of the cut area, as shown in Figure 6. Distilled water, hydrochloric acid, and nitric acid were mixed in a ratio of 4:3:3 to create an etching solution. The samples heated to 650 °C were etched for 20 s, and the samples heated to 1050 °C were etched for 15 s. Afterwards, the microstructure of the samples was evaluated. A Scanning Electron Microscope (SEM) model MIRA4, produced by TESCAN (Brno, Czech Republic), and an Optical Microscope (OM) model BA310 MET, manufactured by MOTIC (Richmond, BC, Canada), were used.

## 3. Results and Discussion

### 3.1. Tensile Properties

**Figure 7:**
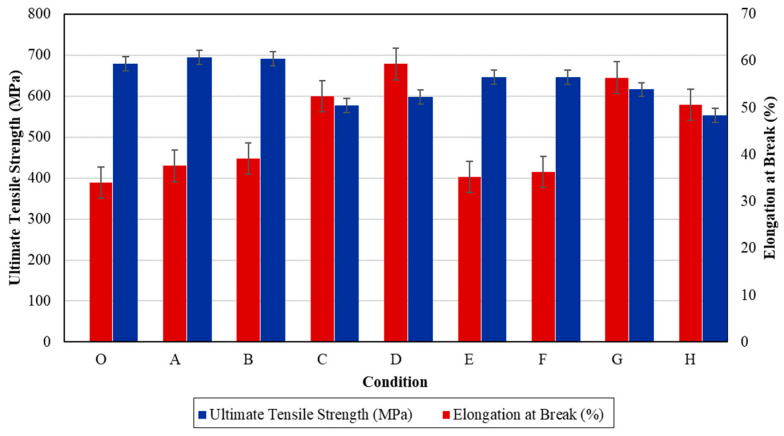
Ultimate tensile strength and elongation at break of each condition.

Tensile properties including ultimate tensile strength (UTS) and elongation at break were evaluated using a universal tensile machine. The values of each condition are displayed in Table 5 and graphed in Figure 7.

According to Table 5 and Figure 7, the ultimate tensile strength (UTS) decreases gradually with increasing temperature. When the workpieces were heated to 1050 °C, UTS was lower, yet the elongation at break was higher compared to the samples heated to 650 °C. The UTS and elongation of the unheated sample (condition O) are 678.34 MPa and 34.07%, respectively. Condition A, in which the specimen was heated to 650 °C for 1 h and quenched in water, has the highest UTS compared to other conditions. The UTS of condition B is slightly higher than that of the unheated sample, at 691.22 MPa. All the heat-treated samples exhibit slightly higher elongation at break compared to the original. However, their UTS is reduced relative to the original, except for conditions A and B. The samples subjected to high-temperature heat treatment at 1050 °C have lower UTS. Condition H has the lowest UTS of 553.07 MPa; however, its elongation at break is quite advanced. Heat treating at 1050 °C causes the UTS to decrease markedly and continuously with time, indicating that both temperature and time significantly affect the mechanical properties. Quenching in air helps reduce UTS compared to quenching in water, pointing out that the quenching medium influences the strength of the specimen. Therefore, the specimens that experienced high baking temperatures exhibit significant deformation, characterized by higher elongation and lower strength. Conversely, when a sample is tempered at the appropriate temperature of 650 °C (conditions A and B), it demonstrates the highest strength value but performs almost the lowest elongation value compared to other conditions. This is attributed to the absence of excessive structural vibration and exposure to extreme temperatures.

The analysis of variance (ANOVA) is a statistical tool used to analyze the average differences in the performance of tests conducted. ANOVA typically employs a tool called the F-test to determine if the design causes any significant changes in quality standards or response values [34]. ANOVA in Table 6 was generated to verify the differences between the three factors and response value which is an ultimate tensile strength (UTS), as well as to ensure the consistency of the model. It observed that a significant factor affecting UTS in the PWHT process for 316 stainless steel is solid solution temperature, since the *p*-value of temperature is 0.003, less than 0.05. Consequently, temperature is a statistically significant parameter in terms of affecting UTS. The two-way and three-way interactions are also demonstrated to be unsubstantial.

### 3.2. Vickers Hardness

**Figure 8:**
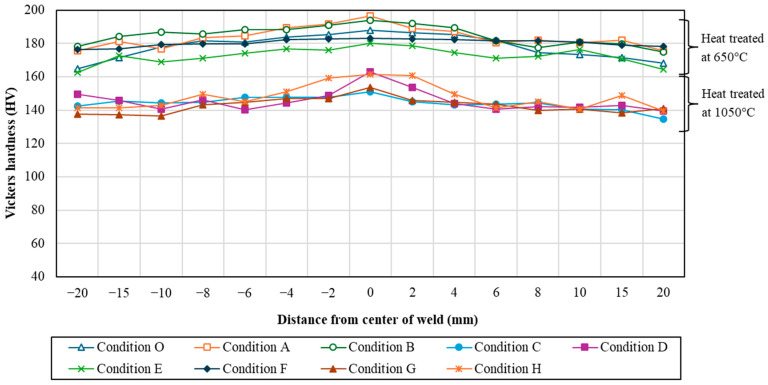
Vicker hardness variations for each compression stage.

The differences in hardness values at each evaluated distance are presented in Table 7. Figure 8 illustrates the graphical variation of hardness in the weldment. Fifteen points were analyzed to explain the hardness of the weld zone, heat-affected zone, and base zone. It is observed that the weld area has the highest hardness value, which gradually decreases along the distance. The lowest value is found in the base zone on the right side of condition C. Additionally, the lowest hardness for every sample is found in the base zone at the point farthest from the center of the weld. Considering the differences influenced by temperature, the hardness values are significantly reduced when heat-treated at higher temperatures (1050 °C) and quenched in both water and air. This indicates that the high temperature, nearly the melting point of 316 stainless steel, is too high to maintain hardness. Among the quenching media, it is found that the trend changes similarly to one another. The highest hardness value is observed in the workpiece heated at 650 °C for an hour, water-quenched, and aged at 500 °C for 24 h, which is caused by precipitation and crystallization. Different phases are exposed in this condition. The heat treatment time also trivially affects the workpiece. However, the most influential factor is temperature, which affects the hardness of the workpiece. It can be concluded that temperature has an important effect on the hardness properties of materials.

The ANOVA for Vickers hardness was summarized in Table 8 to show the consistency between experimental factors and a response value, Vickers hardness. Following Table 8, the significant factors for Vickers hardness are Temperature (A), Time (B), Quench media (C), Temperature*Time (AB), and Temperature*Quench media (AC), as the *p*-value is less than 0.05. Therefore, it can statistically ensure the results in Figure 8 that temperature obviously affects Vickers hardness followed by the quenched media and time, respectively.

### 3.3. Microstructure Observed by Optical Microscope (OM)

#### 3.3.1. OM Images of Base Zone (BZ)

**Figure 9:**
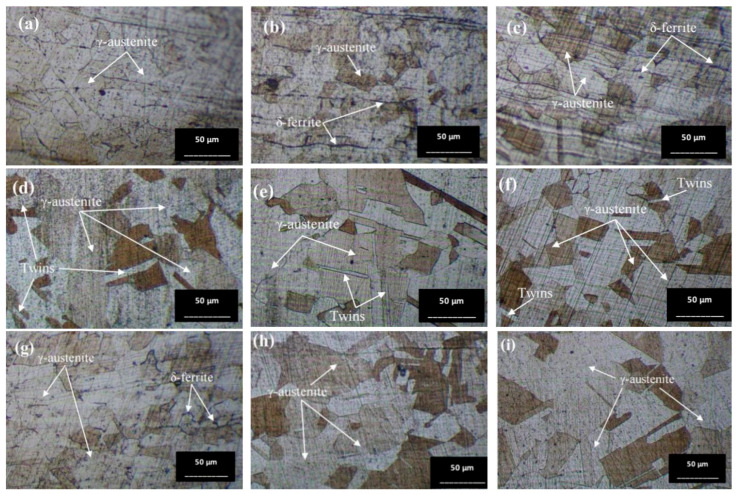
Optical micrographs of base zone (BZ) of (**a**) as-welded, (**b**) condition A, (**c**) condition B, (**d**) condition C, (**e**) condition D, (**f**) condition E, (**g**) condition F, (**h**) condition G, and (**i**) condition H.

The austenite (γ) single phase predominates under nearly all conditions, with grain boundaries becoming more distinctly visible. The grain size shows minimal changes on the surface of the workpieces tempered at 650 °C, appearing slightly larger compared to the as-welded sample. Conversely, the grain size of the workpieces heated to 1050 °C (conditions C, D, G, and H) exhibits a significant increase. Additionally, the base zone of the workpieces tempered at 650 °C contains a few ferrite (δ) phases within the austenite matrix, enhancing the material’s mechanical properties. As seen in Figure 9b, condition A contains the most δ phase compared to the other conditions, which affects the tensile properties and hardness. Furthermore, the surface of the water-quenched workpieces demonstrates only a slight increase in the δ phase.

#### 3.3.2. OM Images of Heat-Affected Zone (HAZ)

**Figure 10:**
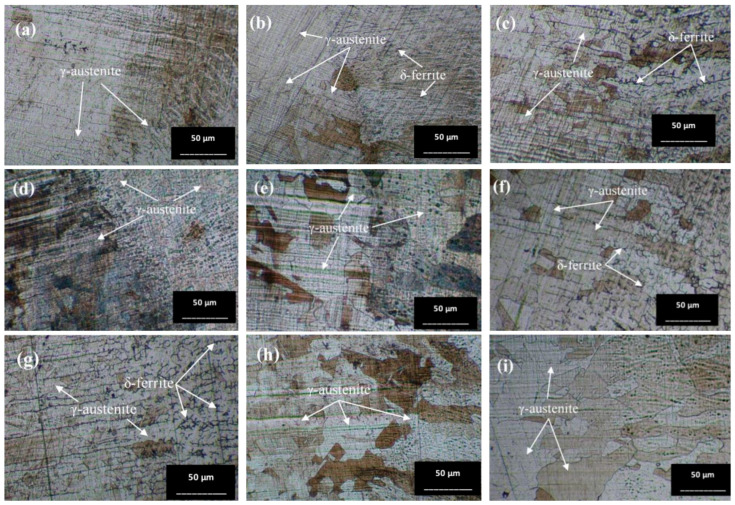
Optical micrographs of heat-affected zone (HAZ) of (**a**) as-welded, (**b**) condition A, (**c**) condition B, (**d**) condition C, (**e**) condition D, (**f**) condition E, (**g**) condition F, (**h**) condition G, and (**i**) condition H.

In the heat-affected zone (HAZ), a phase transformation from γ phase to δ phase is initiated, as evidenced by the presence of some dendritic δ phase, particularly under the conditions of a solid solution temperature of 650 °C (conditions A, B, E, and F), as depicted in Figure 10. Notably, no δ phase is observed in the as-welded sample. The prevalence of the δ phase is higher at 650 °C compared to 1050 °C, especially in condition A. It is observed that the δ phase in the HAZ exhibits a more orderly alignment. Quenching in water results in a finer grain size of the austenite phase. Furthermore, Figure 10 illustrates the grain growth of austenite with increasing time and temperature. Figure 10i demonstrates significant grain growth, attributed to the accelerated thermally assisted migration of the austenite grain boundaries. The high temperature markedly influences the grain size, as evidenced by the comparison between Figure 10c,i.

#### 3.3.3. OM Images of Weld Zone (WZ)

**Figure 11:**
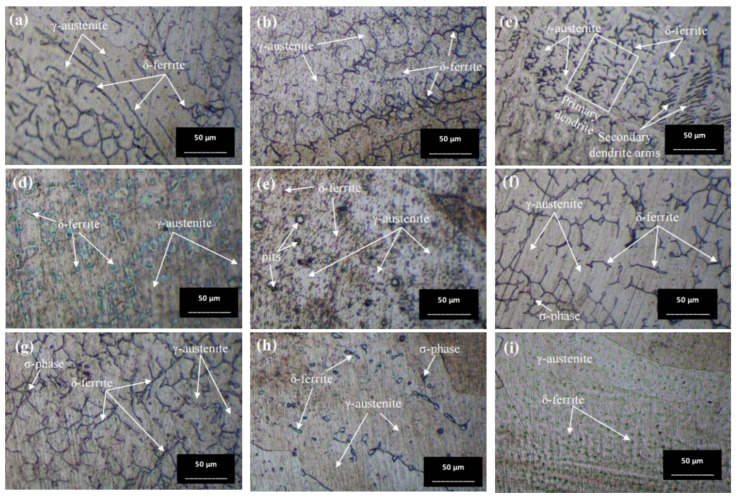
Optical micrographs of weld zone (WZ) of (**a**) as-welded, (**b**) condition A, (**c**) condition B, (**d**) condition C, (**e**) condition D, (**f**) condition E, (**g**) condition F, (**h**) condition G, and (**i**) condition H.

Figure 11 illustrates the microstructure of the welded zone under all conditions. In this weld zone, it was observed that the δ phase decomposed to a two-phase structure of sigma and secondary γ through eutectoid decomposition [35]. A significant portion of the δ phase dissolves in this zone. Figure 11b,c,g,h show obviously the presence of the dendritic δ phase within the austenitic matrix [36]. When tempered at 650 °C, the microstructure exhibits more dendritic branches compared to the as-welded condition (Condition O). The dendritic δ structure suggests that the specimen’s structure at the weld zone is stronger than that of the base zone [37]. This is attributed to the large grainy surface area of each dendrite, where individual branches collide, preventing further crystal formation. Additionally, the amount of δ phase is directly related to the hardness values [38], with Figure 11b or condition A supporting the highest hardness value due to its numerous orderly dendritic δ phases reported previously. Mária Dománková et al. [39] reported that a significant etching of grain boundaries occurs with increased holding time at high temperatures, caused by the precipitation of secondary phases at the grain boundaries. A few particles of the σ-phase were detected at the grain boundaries in several conditions, as indicated by the arrows.

### 3.4. Microstructure Observed by Scanning Electron Microscope (SEM)

**Figure 12:**
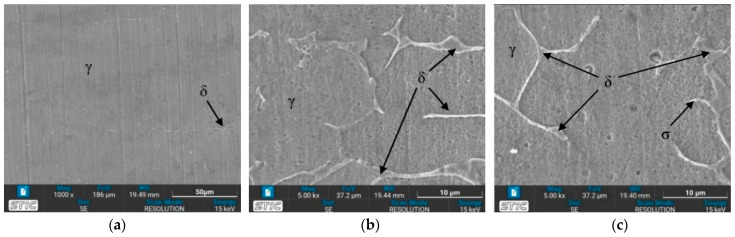
SEM micrographs of as-welded sample, (**a**) BZ, (**b**) HAZ, and (**c**) WZ.

**Figure 13:**
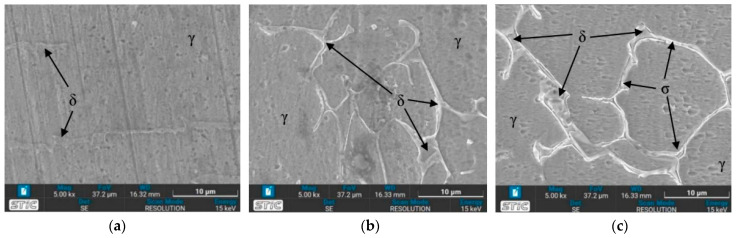
SEM micrographs of sample A, (**a**) BZ, (**b**) HAZ, and (**c**) WZ.

**Figure 14:**
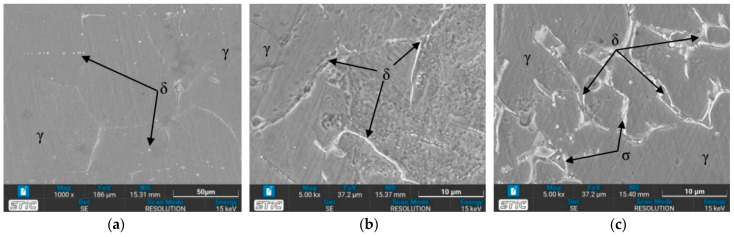
SEM micrographs of sample B, (**a**) BZ, (**b**) HAZ, and (**c**) WZ.

**Figure 15:**
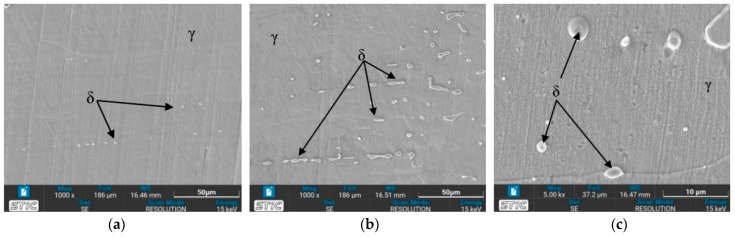
SEM micrographs of sample C, (**a**) BZ, (**b**) HAZ, and (**c**) WZ.

**Figure 16:**
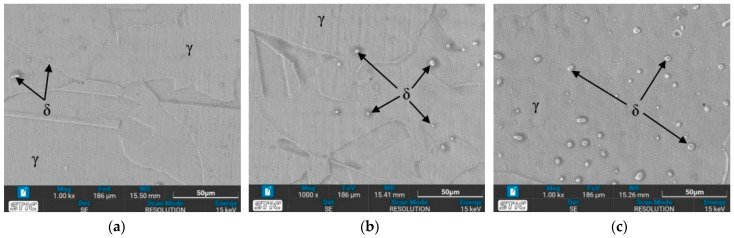
SEM micrographs of sample D, (**a**) BZ, (**b**) HAZ, and (**c**) WZ.

**Figure 17:**
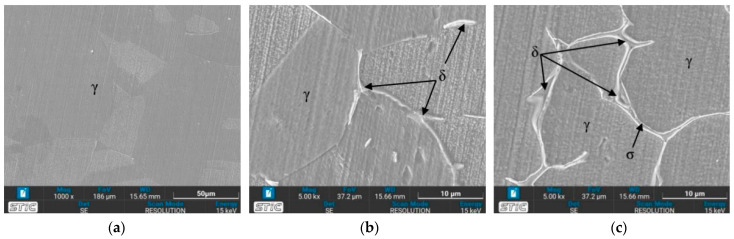
SEM micrographs of sample E, (**a**) BZ, (**b**) HAZ, and (**c**) WZ.

**Figure 18:**
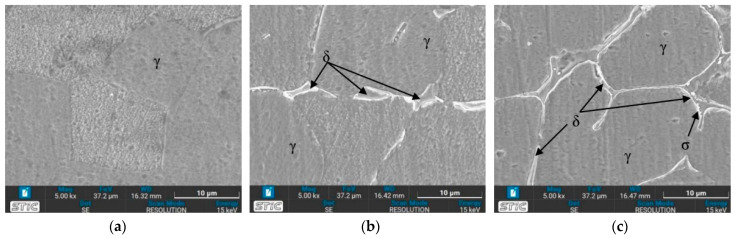
SEM micrographs of sample F, (**a**) BZ, (**b**) HAZ, and (**c**) WZ.

**Figure 19:**
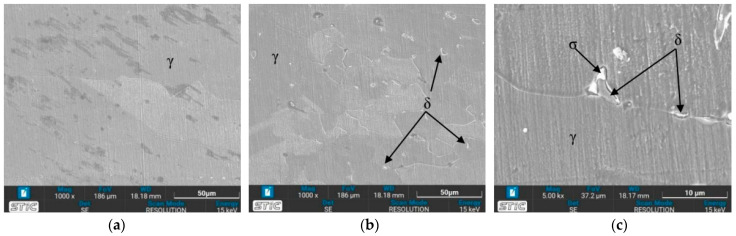
SEM micrographs of sample G, (**a**) BZ, (**b**) HAZ, and (**c**) WZ.

**Figure 20:**
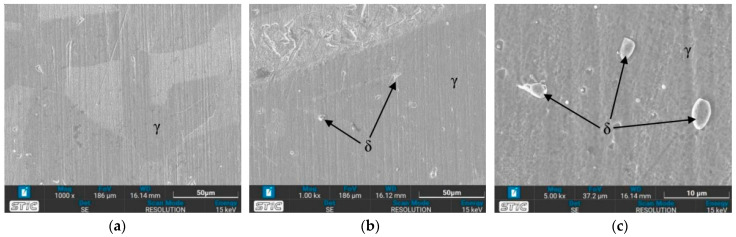
SEM micrographs of sample H, (**a**) BZ, (**b**) HAZ, and (**c**) WZ.

The prepared surfaces were evaluated using a scanning electron microscope (SEM) to determine the texture and size of dendrite grains, assessing their width, length, and branching characteristics. The microstructure observed through SEM exhibited an expanding structure. Images at 1000× and 5000× magnification were utilized, clearly distinguishing the γ, δ, and σ phases. Figure 12, Figure 13, Figure 14, Figure 15, Figure 16, Figure 17, Figure 18, Figure 19 and Figure 20 illustrates the base materials zone (BZ), heat affected zone (HAZ), and welded zone (WZ) of all conditions. These images confirm that each condition resulted in distinct grain changes, particularly in the weld zone (WZ), and that the alloy solidifies primarily as ferrite and then as austenite [40]. For the samples heat-treated at 650 °C, specifically under conditions A, B, E, and F, the dendritic δ phase was more pronounced compared to other conditions. Additionally, the σ phase was observed at the grain boundaries, directly influencing the hardness values. The σ phase forms by the progressive replacement of δ without significant changes in δ phase morphology [41]. According to Chen X. et al. [36], the yield strength (YS), elongation (EL), and hardness are negatively impacted by the presence of σ phases. Ohmura et al. [42] also reported that hardness significantly increases with the precipitation behavior of Laves and σ phases. Consistent with these studies, the hardness and ultimate tensile strength (UTS) of condition A are the highest, attributable to the higher σ phase content in the surface structure. Heat treatment at 650 °C followed by water quenching resulted in larger dendrite grain sizes compared to the as-welded state. In contrast, air quenching produced minimal changes in the transformation.

### 3.5. SEM Micrographs of Fracture Surfaces

**Figure 21:**
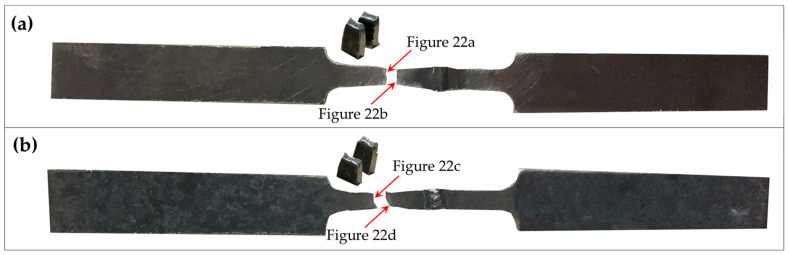
Sample photographs of (**a**) condition A’s specimen and (**b**) condition B’s specimen from the tensile test.

**Figure 22:**
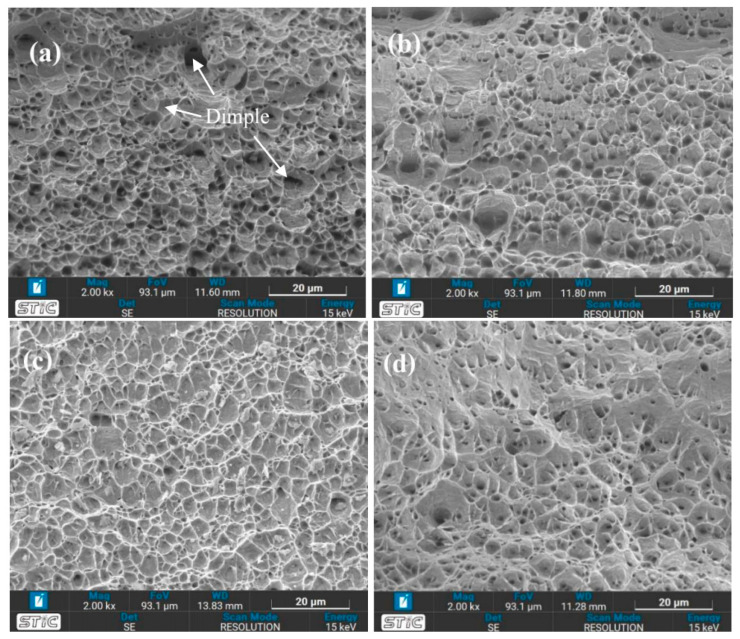
Fracture surfaces after tensile testing of (**a**) condition A (**top**), (**b**) condition A (**bottom**), (**c**) condition H (**top**), and (**d**) condition H (**bottom**).

The results from Table 8 show that condition A, which was heat-treated at 650 °C for one hour, quenched in water, and aged at 500 °C for 24 h, has the highest ultimate tensile strength (UTS). In contrast, condition H has the lowest UTS, with heat treatment at 1050 °C for four hours, followed by air quenching and aging at 500 °C for 24 h. The fracture surfaces of the samples under conditions A and H were analyzed to understand the differences in mechanical properties after the different heat treatments. In Figure 21, the very ductile fracture can be visibly observed for all conditions, with the classic dimple structure being evident. Figure 22 displays the fracture surfaces for the samples under conditions A and H. No non-molten particles are observed in any samples, demonstrating the good consolidation of the parts. Condition A (Figure 22a,b) shows wider and shallower dimple structures, which could explain the lower impact toughness compared to condition H (Figure 22c,d).

## 4. Conclusions

This study examines the optimal parameters of post-weld heat treatment and their effects on the mechanical properties of 316 stainless steel welded using the plasma arc welding process. The primary conclusions are summarized below.

- The ultimate tensile strength of plasma arc-welded 316 stainless steel is significantly influenced by the solid solution temperature. Additionally, the Vickers hardness of plasma arc-welded 316 stainless steel is markedly affected by the solid solution temperature, solid solution duration, and the quenching medium.
- The optimal parameters for the PWHT process of plasma arc-welded 316 stainless steel are a solid solution temperature of 650 °C, a solid solution time of one hour, and water as the quenching medium.
- The highest UTS of 693.93 MPa and Vickers hardness of 196.4 HV at the welded zone were achieved by heat-treating at 650 °C for one hour, quenching in water, and aging at 500 °C for 24 h.
- Heat-treating at 650 °C for one hour, followed by quenching in water and aging at 500 °C for 24 h results in larger dendritic δ grains and contains more σ phase compared to the other conditions, resulting in increased strength and hardness. Additionally, it shows wider and shallower dimple structures, which account for its reduced impact toughness.

## Figures and Tables

**Table 1:** Chemical composition of 316 austenitic stainless-steel plate (wt.%).

Cr	Cu	Mn	Mo	Ni	Al	Si	Fe
16.7225	0.2659	1.5199	2.0981	9.6347	0.5481	0.5209	balance

**Table 2:** The 2^3^ full factorial design of post-weld heat treatment (PWHT) condition.

Factor		Unit	Level	
			Low	High
Temperature	A	°C	650	1050
Time	B	hours	1	4
Quench media	C	-	Water	Air

**Table 3:** Matrix design of PWHT experiment.

Sample	Solid Solution Temperature (°C)	Solid Solution Time (hour)	Quenching Media	Aging
1	650	1	Water	500 °C for 20 h.
2	650	4		
3	1050	1		
4	1050	4		
5	650	1	Air	
6	650	4		
7	1050	1		
8	1050	4		

**Table 4:** Different steps in PWHT process applied in this study.

Heating	Quenching		Aging
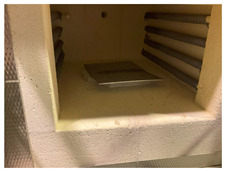	Air quenched (soaked in the oven)	Water quenched	500 °C for 24 h.
	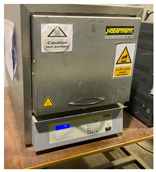	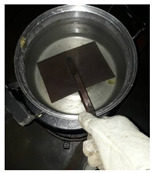	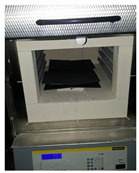

**Table 5:** Ultimate tensile strength (UTS) and elongation at break of each condition.

Condition	Ultimate Tensile Strength (MPa)	Elongation at Break (%)
O = Original (as-weld)	678.34	34.07
A = 650 °C for 1 h, water quenched, age 500 °C for 24 h.	693.93	37.58
B = 650 °C for 4 h, water quenched, age 500 °C for 24 h.	691.22	39.18
C = 1050 °C for 1 h, water quenched, age 500 °C for 24 h.	576.99	52.39
D = 1050 °C for 4 h, water quenched, age 500 °C for 24 h.	597.21	59.32
E = 650 °C for 1 h, air quenched, age 500 °C for 24 h.	645.52	35.18
F = 650 °C for 4 h, air quenched, age 500 °C for 24 h.	645.54	36.27
G = 1050 °C for 1 h, air quenched, age 500 °C for 24 h.	616.09	56.43
H = 1050 °C for 4 h, air quenched, age 500 °C for 24 h.	553.07	50.66

**Table 6:** ANOVA for ultimate tensile strength (UTS) in PWHT process.

Source	DF	Adj. SS	Adj. MS	F-Value	*p*-Value
Model	7	54,788.2	7826.9	5.22	0.003
Temperature	1	41,546.1	41,546.1	27.6	0.000
Time	1	776.4	776.4	0.52	0.482
Quench media	1	3685.5	3685.5	2.46	0.137
2-Way Interactions	3	6007.8	6007.8	1.33	0.298
Temperature*Time	1	603.5	603.5	0.40	0.535
Temperature*Quench media	1	2973.9	2973.9	1.98	0.178
Time*Quench media	1	2430.5	2430.5	1.62	0.221
3-Way	1	2772.4	2772.4	1.85	0.193
Temperature*Time*Quench media	1	2772.4	2772.4	1.85	0.193
Error	16	24,005.1	1500.3		
Total	23	78,793.2			

**Table 7:** Vickers hardness results of each condition.

	BZ (Left)					HAZ		WZ	HAZ		BZ (Right)				
Condition	−20 mm	−15 mm	−10 mm	−8 mm	−6 mm	−4 mm	−2 mm	0 mm	2 mm	4 mm	6 mm	8 mm	10 mm	15 mm	20 mm
O	165.0	171.5	177.9	181.6	180.9	183.9	185.2	187.8	186.6	185.4	181.9	174.6	173.3	171.7	168.1
A	175.8	181.1	176.9	183.4	184.7	189.5	191.6	196.4	189.2	187.3	180.6	182.1	180.4	181.9	175.8
B	178.2	184.3	186.8	185.7	188.2	188.2	190.8	194.0	191.9	189.4	181.7	177.4	180.7	179.6	175.0
C	142.5	145.3	144.3	144.7	147.8	147.8	147.7	151.1	145.1	143.2	143.6	144.2	140.5	140.1	134.7
D	149.7	145.7	140.7	146.2	140.4	144.4	148.9	163.0	153.6	144.1	140.5	142.2	141.7	142.8	139.5
E	162.6	172.6	168.8	171.1	174.1	176.7	176.0	180.1	178.6	174.5	171.2	172.3	176.3	170.9	164.4
F	176.3	176.8	179.5	179.6	179.9	182.2	182.7	183.0	182.8	182.2	181.7	181.6	180.8	179.1	178.2
G	137.8	137.1	136.6	143.2	144.7	147.0	146.8	153.5	145.7	144.6	143.5	139.7	140.5	138.5	141.1
H	141.4	141.3	142.7	149.5	145.2	151.1	159.2	161.3	160.8	149.6	141.5	145.2	140.5	148.9	139.6

**Table 8:** ANOVA for Vickers hardness in PWHT process.

Source	DF	Adj. SS	Adj. MS	F-Value	*p*-Value
Model	7	4288.70	612.67	50.68	0.000
Temperature	1	3730.16	3730.16	308.58	0.000
Time	1	77.88	77.88	6.44	0.035
Quench media	1	143.40	143.40	11.86	0.009
2-Way Interactions	3	100.52	100.52	8.32	0.008
Temperature*Time	1	69.31	69.31	5.73	0.044
Temperature*Quench media	1	231.80	231.80	19.18	0.002
Time*Quench media	1	0.46	0.46	0.04	0.851
3-Way	1	35.70	35.70	2.95	0.124
Temperature*Time*Quench media	1	35.70	35.70	2.95	0.124
Error	8	96.71	12.09		
Total	15				

## Data Availability

Data used in this study are available upon reasonable request to the corresponding author.
